# Dosimetric Evaluation of a Simple Planning Technique for Improving Intensity-Modulated Radiotherapy for Nasopharyngeal Cancer

**DOI:** 10.1371/journal.pone.0129461

**Published:** 2015-07-01

**Authors:** Jia-Yang Lu, Michael Lok-Man Cheung, Mei Li, Bao-Tian Huang, Wen-Jia Xie, Liang-Xi Xie

**Affiliations:** 1 Department of Radiation Oncology, Cancer Hospital of Shantou University Medical College, Shantou, Guangdong, China; 2 Department of Clinical Oncology, Prince of Wales Hospital, Shatin, Hong Kong, China; Van Andel Institute, UNITED STATES

## Abstract

**Purpose:**

To evaluate the dosimetric outcomes of a simple planning technique for improving intensity-modulated radiotherapy (IMRT) for nasopharyngeal cancer (NPC).

**Methods:**

For 39 NPC cases, generally acceptable original plans were generated and were improved by the two planning techniques, respectively: (1) a basal-dose-compensation (BDC) technique, in which the treatment plans were re-optimized based on the original plans; (2) a local-dose-control (LDC) technique, in which the original plans were re-optimized with constraints for hot and cold spots. The BDC, original, and LDC plans were then compared regarding homogeneity index (HI) and conformity index (CI) of planning target volumes (PTVs), organ-at-risk (OAR) sparing and monitor units (MUs) per fraction. The whole planning times were also compared between the BDC and LDC plans.

**Results:**

The BDC plans had superior HIs / CIs, by 13-24% / 3-243%, respectively, over the original plans. Compared to the LDC plans, the BDC plans provided better HIs only for PTVnx (the PTV of nasopharyngeal primary tumor) by 11% and better CIs for all PTVs by 2-134%. The BDC technique spared most OARs, by 1-9%. The average MUs of the BDC, original, and LDC plans were 2149, 2068 and 2179, respectively. The average whole planning times were 48 and 69 minutes for the BDC and LDC plans, respectively.

**Conclusions:**

For the IMRT of nasopharyngeal cancer, the BDC planning technique can improve target dose homogeneity, conformity and OAR sparing, with better planning efficiency.

## Introduction

Nasopharyngeal cancer (NPC) is a common malignant head and neck tumor in southern China and Southeast Asia [1,2]. Over the last decade, intensity-modulated radiotherapy (IMRT) has become the mainstay in the treatment of non-metastatic NPC [3]. IMRT combines several intensity-modulated beams to obtain improved dose homogeneity and highly conformal dose distributions, as well as improved normal-structure sparing. However, IMRT planning for NPC is challenging due to the complex anatomy, with bones, soft tissues and air cavities all in need of consideration. Moreover, organs at risk (OARs), such as spinal cord, brainstem, and parotid glands, are typically located proximately to the target volumes. In addition, the targets are prescribed at different dose levels [4] and the target volumes often have irregular concave shapes [5].

For NPC IMRT planning, a number of planning techniques have been reported before. Chau et al [6] and Zhang et al [7] introduced two split-organ approaches, respectively to reduce the doses of parotids in the NPC IMRT planning. These planning techniques only considered the trade-offs between the parotids and other organs, because the doses to other OARs were increased though not significantly. Cheng et al [8] and Budrukkar et al [9] focused on beam arrangement and number for the IMRT planning, respectively. Although selecting the optimal arrangement and number of beams is an effective approach, it is still difficult to achieve the optimal plans because of a systematic error called optimization-convergence error (OCE) [10,11], which may result in differences between the optimizer plans and the finally calculated deliverable plans. The OCE would inevitably arise in the IMRT planning using current treatment planning systems, because up to now, the treatment planning computer is not fast enough for the optimizer to use a full volume dose calculation algorithm for routine optimizing, but use a simplified algorithm instead. Due to the OCE, the finally calculated dose distribution may not meet the objectives after the completion of optimization process, although the dose distribution in the optimizer has already met them.

Accordingly, we proposed a planning technique named basal-dose-compensation (BDC) technique to improve the IMRT plans for NPC, by means of compensating for the OCE utilizing a “base dose plan”. To assess the efficacy of this new technique, we utilized the original plans for longitudinal comparison, and another common planning technique that was called local-dose-control (LDC) technique applied for controlling the local doses of hot and cold spots [12,13], for lateral comparison.

## Materials and Methods

### Ethics Statement

The protocol was approved by the Ethical Commission of the Cancer Hospital of Shantou University Medical College. Because this was not a treatment-based study, our institutional review board waived the need for written informed consent from the participants. The patient information was anonymized and de-identified to protect patient confidentiality.

### Patient characteristics

Thirty-nine newly-diagnosed, previously-untreated and non-metastatic NPC patients were retrospectively identified. The patients included 35 males and 4 females, with an age range of 24–68 years (median, 47 years). In accordance with the American Joint Committee on Cancer (AJCC) Seventh Edition staging system, the tumor stages of patients were described as follows (T1–T4, N1–N3 and M0): Stage II, 2; Stage III, 16; Stage IV, 21.

### CT simulation

All patients were immobilized in the supine position in a tailor-made thermoplastic cast from head to shoulders. CT scans with intravenous contrast using a 3-mm slice thickness from the head to 2 cm below the sternoclavicular joint were performed by a 16-slice CT scanner (Philips Brilliance CT Big Bore Oncology Configuration, Cleveland, OH, USA). The CT images were then transferred to the Eclipse version 10.0 treatment planning system (Varian Medical System, Inc., Palo Alto, CA, USA) for target and OAR delineation and treatment planning.

### Target delineation and OAR definition

All target volumes were delineated by our radiation oncologists. Targets and the OARs were localized on the basis of the CT images and modified according to the pre-treatment MRI images in fusion. The nasopharyngeal gross tumor volume (GTVnx) included all known primary tumor gross disease and retropharyngeal lymphadenopathy, as determined by the CT images, MRI images, and endoscopic findings. Enlarged positive neck lymph nodes were localized as a separate gross tumor volume (GTVnd). CTV60 was defined as the clinical target volume at high risk for involvement, including GTVnx, GTVnd, the entire nasopharynx, retropharyngeal nodal regions, skull base, clivus, pterygoid fossae, parapharyngeal space, sphenoid sinus, the posterior one-third of the nasal cavity, maxillary sinuses, part of the posterior ethmoid sinus, and the electively prophylactic irradiated cervical nodal regions. The planning target volumes (PTVs), which included PTVnx, PTVnd and PTV60, were generated by 5-mm outer margins of GTVnx, GTVnd and CTV60, respectively. In order to evaluate the dose homogeneity of PTV60 with the avoidance of the higher doses of PTVnx and PTVnd, PTV60_only was defined as the PTV60 minus 1 cm expansion volumes of both PTVnx and PTVnd.

The OARs, including the spinal cord, brainstem, lenses, optic nerves, optic chiasm, larynx, oral cavity and parotid glands, were delineated on the CT images. Planning organ-at-risk volumes (PRVs) were created for spinal cord and brainstem by adding 5-mm and 3-mm margins to them, respectively, and denoted as “PRV spinal cord” and “PRV brainstem”, respectively. Normal tissue was defined as the body volume excluding all the PTVs.

### IMRT planning

Nine coplanar fields of 6-MV photon beams from a Truebeam (Varian Medical System, Inc., Palo Alto, CA) linear accelerator were generated for each plan in Eclipse. The fields were placed at evenly distributed gantry angles, 40° apart, at 200°, 240°, 280°, 320°, 0°, 40°, 80°, 120° and 160°. Dose-limiting ring structures were generated to form dose gradients surrounding the PTVs. The Dose Volume Optimizer (DVO, version 10.0.28) algorithm and Anisotropic Analytical Algorithm (AAA, version 10.0.28) were applied for optimization and final dose calculations, with a grid size of 2.5 mm. Dose prescribing was as follows: 70.4 Gy (2.2 Gy/fraction × 32 fractions) for PTVnx, 66 Gy (2.06 Gy/fraction × 32 fractions) for PTVnd, 60 Gy (1.88 Gy × 32 fractions) for PTV60. Each treatment plan was normalized to the 70.4-Gy prescribed dose covering 95% of PTVnx.

The planning constraints for the PTVs and OARs were shown in Table 1. All the plans were designed to meet the planning constraints except for the advanced cases in which making compromises were necessary. The PTV coverage objectives were set to the highest priorities, followed by the OAR sparing. D_x%_ represents the dose which is reached or exceeded in x% of the volume and V_100%_ represents the % volume receiving at least 100% of the prescription dose. D_max_ represents the maximum dose, and D_mean_ represents the mean dose. The D_mean_ values of the larynx, oral cavity and parotids were limited by the “upper objective” options.

**Table 1:** Treatment planning constraints.

Structure(s)	Planning constraint(s)
**PTVnx**	V_100%_ = 95%, D_max_ < 77.44 Gy
**PTVnd**	V_100%_ ≥ 95%
**PTV60**	V_100%_ ≥ 95%
**Spinal cord**	D_max_ < 45 Gy
**PRV spinal cord**	D_max_ < 50 Gy or V_50Gy_ < 1%
**Brainstem**	D_max_ < 54 Gy or D_max_ < 60 Gy
**PRV brainstem**	D_max_ < 60 Gy or V_60Gy_ < 1%
**Lenses**	D_max_ < 10 Gy
**Optic nerves/chiasm**	D_max_ < 54 Gy or D_max_ < 60 Gy
**Larynx**	D_mean_ < 40 Gy
**Oral cavity**	D_mean_ < 40 Gy
**Parotids**	D_mean_ < 40 Gy
**Normal tissue**	As low as possible

PTVnx, planning target volume of nasopharyngeal primary tumor; PTVnd, planning target volume of positive lymph nodes; PTV60, planning target volume receiving a prescribed dose of 60 Gy; PRV, planning organ-at-risk volume; V_x%_, % volume receiving at least x% of prescription dose; V_xGy_, % volume receiving at least x Gy dose; D_max_, maximum dose; D_mean_, mean dose.

For generating the original plan, the optimization objectives of each plan were adjusted until the plan was clinically acceptable. Then two copies of the original plan were improved by the two techniques below, respectively, with the original optimization objectives unmodified: (1) BDC planning technique (BDC plan) and (2) LDC technique (LDC plan).

To generate a BDC plan, the number of fractions of the original plan was changed to 50% of the prescribed number of fractions (from 32 to 16 in our cases) in order to generate a base dose plan with half of the total prescription dose. Then the base dose plan was copied to generate a “top dose plan”. Then, this top dose plan was re-optimized based on the base dose plan employing Eclipse’s “base dose plan” function with 20 maximum iterations. In this situation, the prescription dose of the plan sum (the top dose plan plus the base dose plan) was equal to the original prescription dose. When the final dose calculation was completed, the number of fractions of the optimized top dose plan was restored from 50% (16 fractions) to 100% (32 fractions) of the prescribed number of fractions, that is, the prescription dose of the top dose plan was changed from a half to the original total. The resultant optimized top dose plan was the BDC plan.

To generate a LDC plan, the volumes of 105% of prescription isodoses of all the PTVs were converted to hot-spot structures, which were added as upper objectives that were set to 1–4% lower than the prescription doses. The volumes of isodose of ≥ 50 Gy in PRV brainstem and the isodose of ≥ 40 Gy in PRV spinal cord were converted to hot-spot structures as upper objectives, which were set to 50 Gy and 40 Gy, respectively to reduce the D_max_ of them. By subtracting the prescription isodose volumes (PIVs) from the PTVs, the cold-spot structures were generated, and were set to 1% higher than the prescription doses. After additional optimization objectives were assigned, the plan was re-optimized with 20 maximum iterations, and lastly, the LDC plan was completed.

All the plans were conducted by the same medical physicist to avoid individual variation. Distributed calculation framework (DCF) was employed for accelerating final dose calculation process. The whole planning times taking the original planning times into consideration were recorded and compared between the BDC and LDC plans. The monitor units (MUs) per fraction for each plan were also compared.

### Plan evaluation

Dose-volume statistics, isodose distributions, and cumulative dose-volume histograms (DVHs) were computed and compared among the three types of plans. D_2%_ and D_98%_ were selected [14] as near-maximal and near-minimal doses, respectively for the PTV, for evaluating hot and cold spots. The target dose homogeneity was measured with homogeneity index (HI), which was defined by the formula below:

$$HI=\frac{D_{2\%}-D_{98\%}}{D_{50\%}}$$

A conformity index (CI) [15] which accounting for the overlap between target volume (TV) and PIV was used to measure the target dose conformity and was defined by the formula below:

$$CI=\frac{{\left(TV\;within\;PIV\right)}^{2}}{TV\times PIV}$$

The HI value was between 0 and 1, with 0 representing the ideal homogeneity, whereas the CI value was between 0 and 1, with 1 representing the ideal conformity. All the evaluation indicators used for PTVs and OARs are summarized in Table 2.

**Table 2:** Evaluation indicators for the treatment plans.

Structure(s)	Evaluation indicator(s)
**PTVnx**	D_2%_, D_98%_, D_50%_, V_100%_, HI, CI,
**PTVnd**	D_2%_, D_98%_, D_50%_, V_100%_, HI, CI,
**PTV60**	D_98%_, D_50%_, V_100%_, CI
**PTV60_only**	D_2%_, HI
**Spinal cord**	D_max_, D_mean_
**PRV spinal cord**	D_max_, D_mean_
**Brainstem**	D_max_, D_mean_
**PRV brainstem**	D_max_, D_mean_
**Lenses**	D_max_
**Optic nerves**	D_max_
**Optic chiasm**	D_max_
**Larynx**	D_mean_
**Oral cavity**	D_mean_
**Parotids**	D_mean_
**Normal tissue**	D_mean_

PTVnx, planning target volume of nasopharyngeal primary tumor; PTVnd, planning target volume of positive lymph nodes; PTV60, planning target volume receiving a prescribed dose of 60 Gy; PTV60_only, the PTV60 minus 1 cm expansion volumes of both PTVnx and PTVnd; PRV, planning organ-at-risk volume; D_x%_, dose which is reached or exceeded in x% of the volume; V_100%_, % volume receiving at least 100% of the prescription dose; HI, homogeneity index; CI, conformity index; D_max_, maximum dose; D_mean_, mean dose.

### Statistical analysis

To determine the statistical significance of the differences between the BDC and original plans, as well as the differences between the BDC and LDC plans, two-tailed paired Wilcoxon signed-rank tests were performed with *p*-value of < 0.05 considered to be significant, using SPSS version 19 software (SPSS, Inc., Chicago, IL, USA).

## Results

All the plans improved by the two planning techniques met the requirement that the 100% of prescription doses covered at least 95% of the PTVs with acceptable maximum doses. The doses of most OARs were below the tolerance limits, except in some advanced cases, where the D_max_ of ipsilateral lens or optic nerve as well as the D_mean_ of ipsilateral parotid, oral cavity or larynx exceeded the tolerance dose limits.

### Target dose homogeneity and conformity

**Fig 1:**
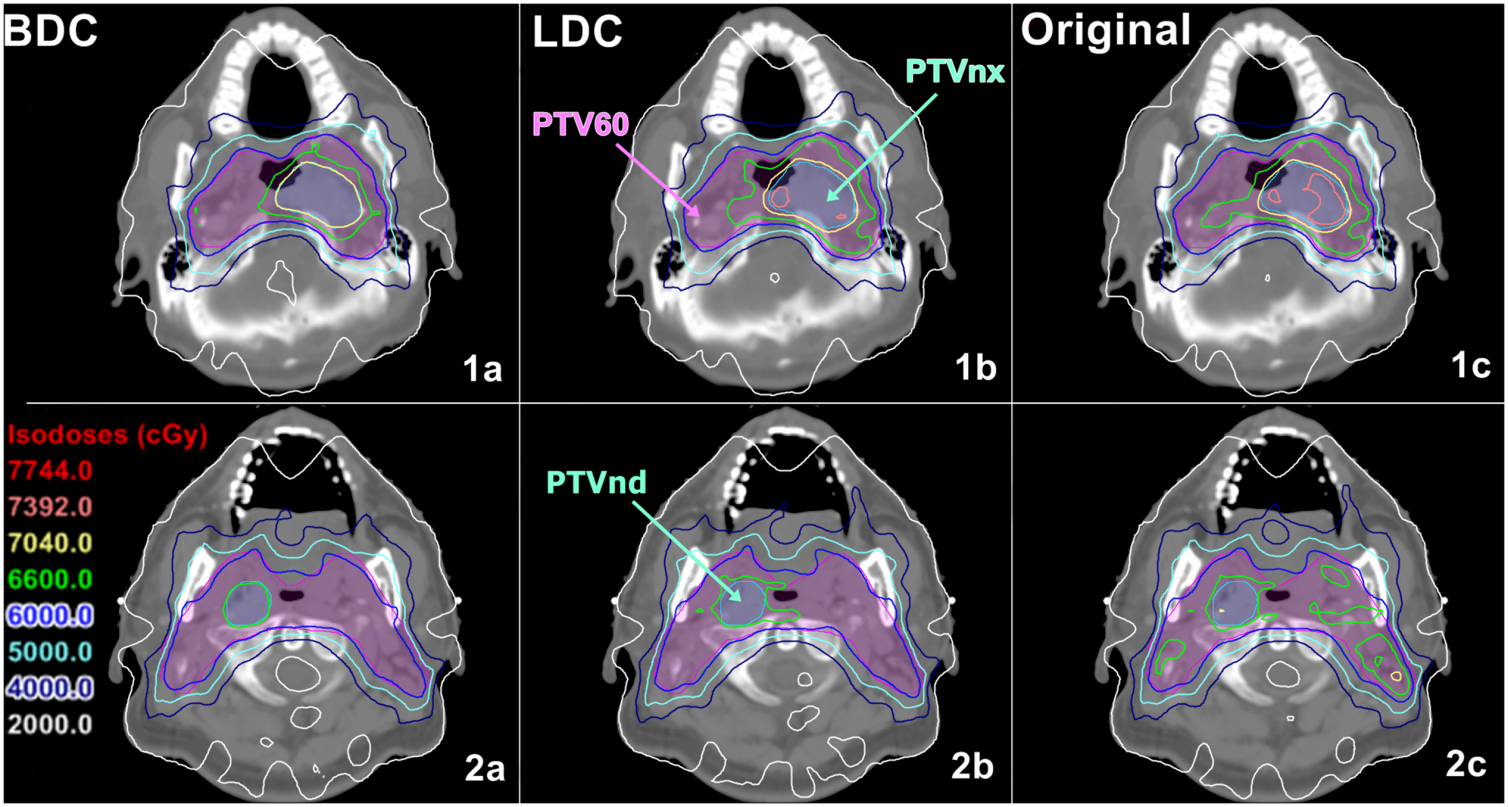
Dose distributions of the BDC, original and LDC plans for two representative cases (case 1: 1a, 1b, 1c; case 2: 2a, 2b, 2c).

**Fig 2:**
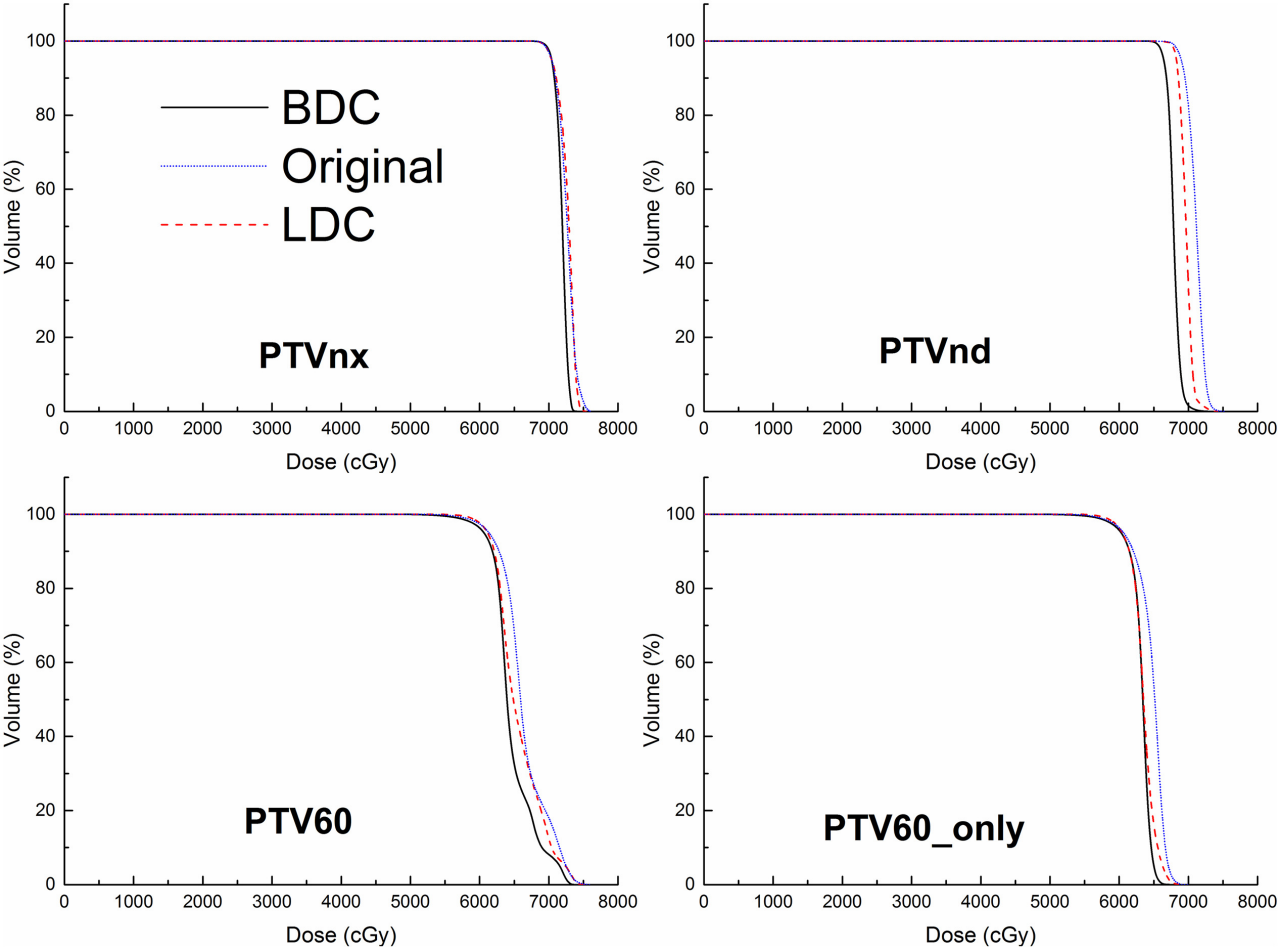
Dose-volume histograms (DVHs) of the planning target volumes (PTVs) for the BDC, original and LDC plans in one representative case.

**Fig 3:**
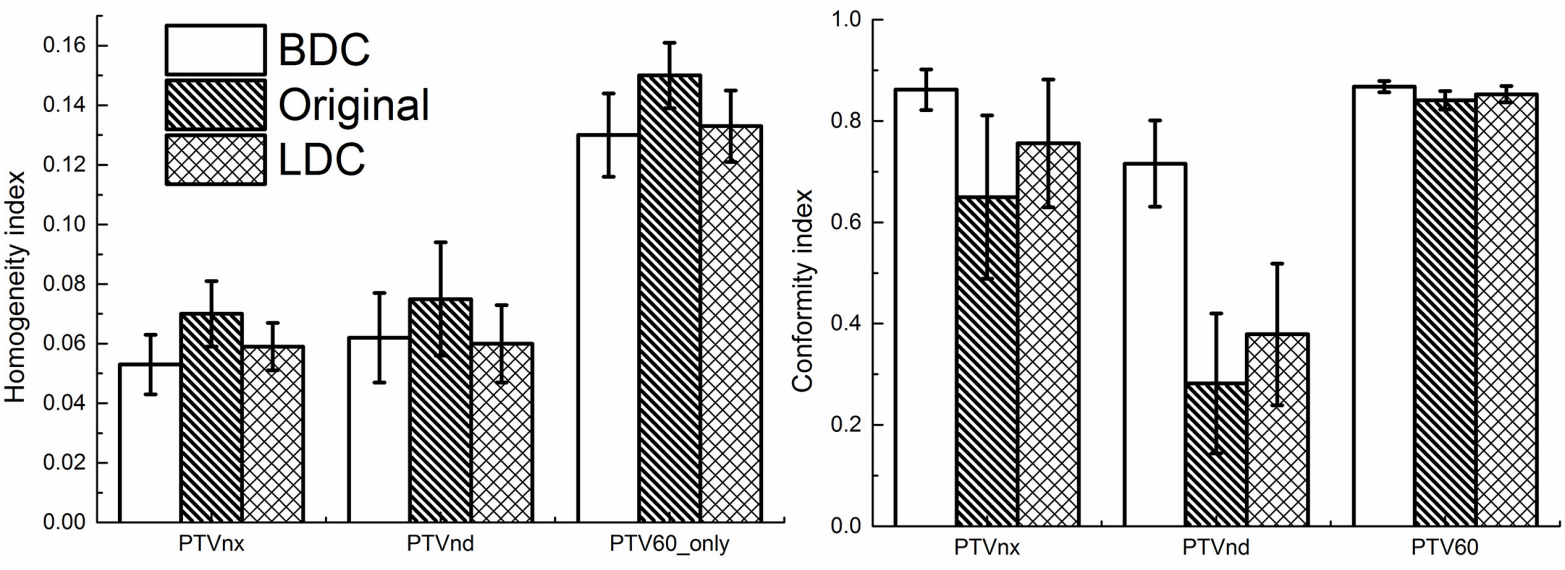
Average homogeneity index (HI) and average conformity index (CI) of the planning target volumes (PTVs) for the BDC, original and LDC plans.

As summarized in Table 3, the BDC plans provided superior target dose homogeneity and conformity over the other two plans. the D_2%_ of PTVnx in the BDC plans was significantly lower than those of the original and LDC plans (by 1.7% and 0.7%, respectively), while there was no significant difference for the D_98%_ of PTVnx. In terms of the HI, the BDC plans were significantly better than the original plans for PTVnx (by 23.8%), PTVnd (by 15.1%) and PTV60_only (by 13.2%), whereas the BDC plans were significantly better than the LDC plans only for PTVnx (by 10.6%). With regards to the CI, the BDC plans were significantly better than the original and LDC plans for PTVnx (by 42.2% and 17.6%, respectively), PTVnd (by 242.6% and 133.7%, respectively) and PTV60 (by 3.3% and 1.8%, respectively). For the isodose distributions, significantly fewer hot spots were observed in the BDC plans, and the isodose lines appeared more conformal to the PTVs (Fig 1). The DVH curves of the PTVs were steeper for the BDC plans indicating more homogeneous dose distributions (Fig 2). Fig 3 shows the average homogeneity index and conformity index for the PTVs within the three plans.

**Table 3:** Dosimetric parameters of the planning target volumes (PTVs) for the BDC, original and LDC plans.

		BDC	Original	LDC	p-value	
					BDC vs. Original	BDC vs. LDC
**PTVnx**	**D**_**2%**_ **(Gy)**	73.49 ± 0.47	74.77 ± 0.77	74.00 ± 0.45	0.000	0.000
	**D**_**98%**_ **(Gy)**	69.70 ± 0.28	69.73 ± 0.18	69.74 ± 0.28	0.234	0.135
	**D**_**50%**_ **(Gy)**	72.04 ± 0.23	72.49 ± 0.36	72.31 ± 0.31	0.000	0.000
	**V**_**100%**_ **(%)**	95.0 ± 0.0	95.0 ± 0.0	95.0 ± 0.0	1.000	1.000
	**HI**	0.053 ± 0.010	0.070 ± 0.011	0.059 ± 0.008	0.000	0.000
	**CI**	0.862 ± 0.040	0.650 ± 0.161	0.756 ± 0.126	0.000	0.000
**PTVnd**	**D**_**2%**_ **(Gy)**	70.16 ± 0.88	72.62 ± 1.21	71.25 ± 1.15	0.000	0.000
	**D**_**98%**_ **(Gy)**	65.92 ± 0.34	67.36 ± 1.25	67.08 ± 0.98	0.000	0.000
	**D**_**50%**_ **(Gy)**	68.15 ± 0.36	70.47 ± 0.94	69.48 ± 1.01	0.000	0.000
	**V**_**100%**_ **(%)**	97.5 ± 1.3	99.2 ± 1.2	99.2 ± 1.0	0.000	0.000
	**HI**	0.062 ± 0.015	0.075 ± 0.019	0.060 ± 0.013	0.000	0.159
	**CI**	0.716 ± 0.085	0.282 ± 0.138	0.379 ± 0.140	0.000	0.000
**PTV60**	**D**_**98%**_ **(Gy)**	58.11 ± 0.53	59.34 ± 0.60	59.55 ± 0.45	0.000	0.000
	**D**_**50%**_ **(Gy)**	64.31 ± 0.53	66.33 ± 0.85	65.53 ± 1.06	0.000	0.000
	**V**_**100%**_ **(%)**	95.7 ± 0.6	97.3 ± 0.7	97.2 ± 0.8	0.000	0.000
	**CI**	0.868 ± 0.011	0.841 ± 0.018	0.853 ± 0.016	0.000	0.000
**PTV60_only**	**D**_**2%**_ **(Gy)**	65.65 ± 0.42	68.31 ± 0.83	67.32 ± 0.78	0.000	0.000
	**HI**	0.130 ± 0.014	0.150 ± 0.011	0.133 ± 0.012	0.000	0.151

BDC, basal dose compensation; LDC, local dose control; PTVnx, planning target volume of nasopharyngeal primary tumor; PTVnd, planning target volume of positive lymph nodes; PTV60, planning target volume receiving a prescribed dose of 60 Gy; PTV60_only, the PTV60 minus 1 cm expansion volumes of both PTVnx and PTVnd; D_x%_, dose which is reached or exceeded in x% of the volume; V_100%_, % volume receiving at least 100% of the prescription dose; HI, homogeneity index; CI, conformity index.

### OAR sparing

**Fig 4:**
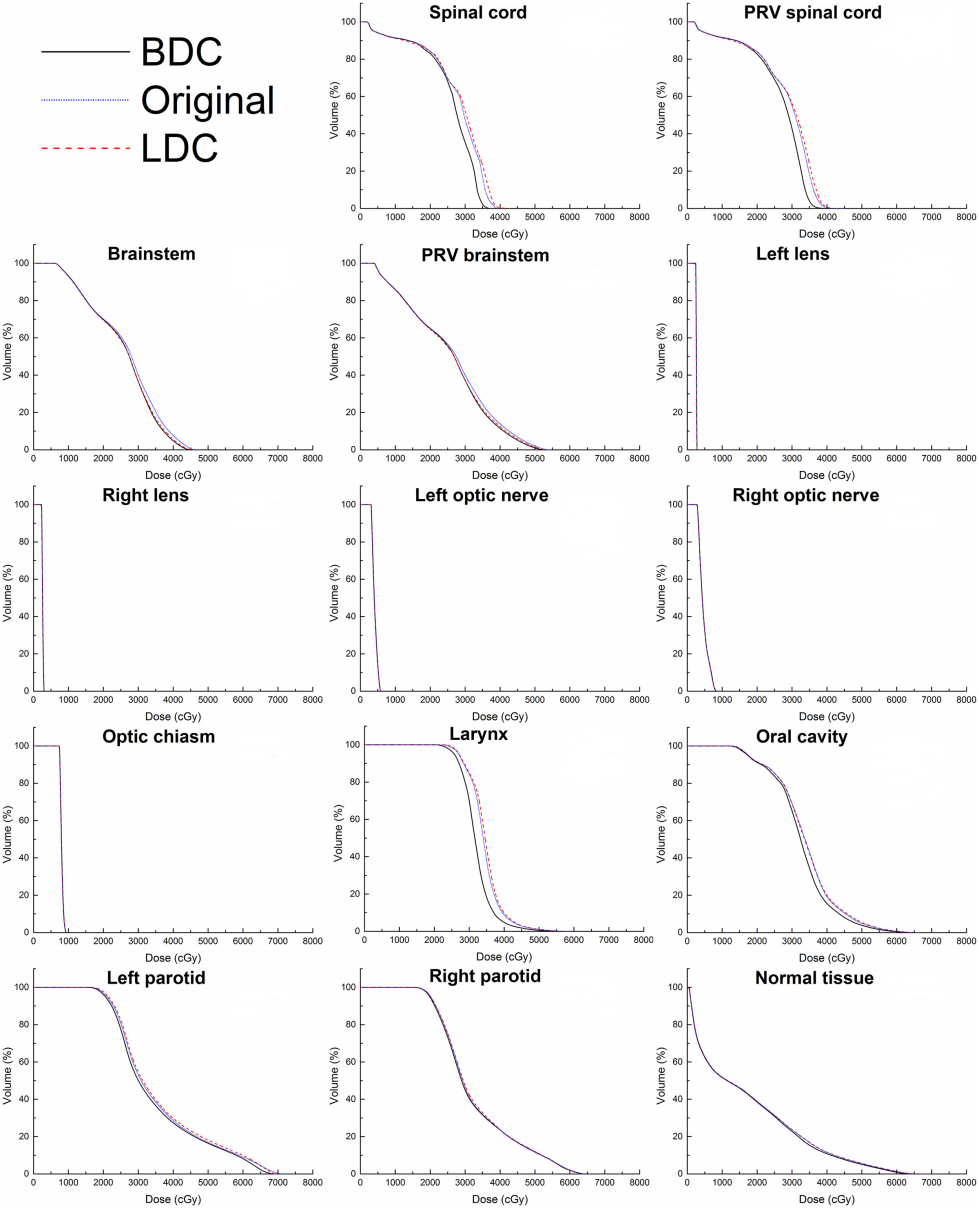
Dose-volume histograms (DVHs) of the organs at risk (OARs) for the BDC, original and LDC plans in one representative case.

As shown in Table 4, the BDC plans tended to deposit lower doses in most OARs. Compared to the original plans, the BDC plans demonstrated significantly lower D_max_ of the spinal cord (by 6.3%), PRV spinal cord (by 3.3%), brainstem (by 2.6%), PRV brainstem (by 2.0%) and left lens (by 1.2%), as well as significantly lower D_mean_ of the spinal cord (by 6.2%), PRV spinal cord (by 6.1%), brainstem (by 2.7%), PRV brainstem (by 2.5%), larynx (by 7.3%), oral cavity (by 3.2%), left parotid (by 3.8%), right parotid (by 3.2%) and normal tissue (by 2.1%). When compared to the LDC plans, the BDC plans demonstrated significantly lower D_max_ of the spinal cord (by 8.2%), left lens (by 1.5%) and right lens (by 0.6%), as well as lower D_mean_ of the spinal cord (by 6.6%), PRV spinal cord (by 6.3%), brainstem (by 1.3%), PRV brainstem (by 1.3%), larynx (by 7.4%), oral cavity (by 4.1%), left parotid (by 8.5%), right parotid (by 7.9%) and normal tissue (by 2.1%), but no significant differences were identified for the D_max_ of PRV spinal cord, brainstem and PRV brainstem. Moreover, there were no significant differences with respect to the D_max_ of the optic chiasm and optic nerves among the three plans. Fig 4 shows the DVHs of the OARs among the three different plans in one representative case.

**Table 4:** Dosimetric parameters of the organs at risk for the BDC, original and LDC plans.

		BDC	Original	LDC	p-value	
	(Gy)				BDC vs. Original	BDC vs. LDC
**Spinal cord**	**D**_**max**_	38.11 ± 2.54	40.68 ± 2.70	41.53 ± 2.24	0.000	0.000
	**D**_**mean**_	24.51 ± 2.15	26.15 ± 2.56	26.26 ± 2.49	0.000	0.000
**PRV spinal cord**	**D**_**max**_	47.18 ± 5.97	48.76 ± 5.76	47.32 ± 4.91	0.000	0.727
	**D**_**mean**_	24.89 ± 2.13	26.52 ± 2.50	26.57 ± 2.44	0.000	0.000
**Brainstem**	**D**_**max**_	49.78 ± 3.93	51.11 ± 3.98	49.89 ± 3.80	0.000	0.468
	**D**_**mean**_	26.44 ± 6.05	27.16 ± 6.19	26.78 ± 6.14	0.000	0.000
**PRV brainstem**	**D**_**max**_	58.33 ± 3.46	59.51 ± 3.69	58.11 ± 3.58	0.000	0.298
	**D**_**mean**_	26.99 ± 6.26	27.68 ± 6.39	27.34 ± 6.34	0.000	0.000
**Left lens**	**D**_**max**_	6.34 ± 2.55	6.42 ± 2.59	6.46 ± 2.64	0.001	0.004
**Right lens**	**D**_**max**_	6.45 ± 2.86	6.43 ± 2.76	6.49 ± 2.80	0.365	0.032
**Left optic nerve**	**D**_**max**_	32.42 ± 20.65	32.30 ± 20.53	32.48 ± 20.76	0.950	0.451
**Right optic nerve**	**D**_**max**_	32.23 ± 20.29	32.11 ± 20.25	32.44 ± 20.53	0.596	0.346
**Optic chiasm**	**D**_**max**_	30.32 ± 18.16	30.34 ± 18.21	30.62 ± 18.53	0.911	0.053
**Larynx**	**D**_**mean**_	34.56 ± 2.08	37.27 ± 2.16	37.30 ± 2.15	0.000	0.000
**Oral cavity**	**D**_**mean**_	34.82 ± 4.07	35.94 ± 4.00	36.30 ± 4.06	0.000	0.000
**Left parotid**	**D**_**mean**_	35.25 ± 2.04	36.66 ± 2.12	38.58 ± 2.41	0.000	0.000
**Right parotid**	**D**_**mean**_	35.12 ± 2.88	36.29 ± 2.80	38.23 ± 3.88	0.000	0.000
**Normal tissue**	**D**_**mean**_	16.56 ± 1.80	16.92 ± 1.86	16.92 ± 1.85	0.000	0.000

BDC, basal dose compensation; LDC, local dose control; PRV, Planning organ-at-risk volume; D_max_, maximum dose; D_mean_, mean dose.

### Planning time and MUs

As shown in Table 5, it took 28.0% less time to complete a treatment plan with the BDC technique than with the LDC technique. However, the MUs of BDC plans were 4.1% more than those of original plans while the MUs of the BDC plans were 1.3% fewer than those of the LDC plans.

**Table 5:** Planning time and monitor units for the BDC, original and LDC plans.

	BDC	Original	LDC	p-value	
				BDC vs. Original	BDC vs. LDC
**Planning time (minute)**	48 ± 10	NA	69 ± 14	NA	0.000
**Monitor units**	2149 ± 227	2068 ± 249	2179 ± 233	0.000	0.044

BDC, basal dose compensation; LDC, local dose control; NA, not applicable.

## Discussion

NPC is one of the cancers of which IMRT plays an important role in the treatment [16,17]. It is essential to improve the planning technique to give full scope to the advantages of IMRT for NPC, that is, to achieve better target dose homogeneity, conformity and better OAR sparing.

The most obvious advantage of the BDC technique is to improve the dose homogeneity. The BDC technique can significantly improve the dose homogeneity for all the PTVs when compared to the original plans, and for the PTVnx when compared to the LDC technique. The improved uniform dose distribution may result in a potential clinical benefit, because the PTVs of NPC commonly contain such tissues as the mucosa and submucosal tissues, nerves and bones, which may suffer complications after receiving significantly heterogeneous high doses [18].

The BDC technique demonstrated better conformity, which can better spare the surrounding healthy tissue. It could further reduce 1–9% of the dose delivered to most of the OARs including the (PRV) spinal cord, (PRV) brainstem, larynx, oral cavity, parotids and normal tissue.

The reductions of the doses delivered to (PRV) spinal cord and (PRV) brainstem were expected to reduce the risks of radiation-induced myelitis and brainstem necrosis [19]. It could be beneficial to the NPC patients with locally residual or recurrent diseases, especially when re-irradiation is required [3].

One advantage of the IMRT for NPC lies in parotid function preservation [20,21]. The researches performed by Hsiung et al [22] and Kwong et al [23] revealed the close relationship between mean parotid dose and parotid function. As known to all, xerostomia caused by parotid gland dysfunction may contribute to dental decay, oral infections, fissures, and dysphagia and is one of the most prevalent factors affecting the quality of life of post-radiotherapy patients [18]. Our study demonstrated that the BDC technique could reduce the mean dose delivered to the parotid glands by approximately 1–3 Gy without compromising tumor coverage, thus it may reduce the incidence of xerostomia. Besides, the mean dose to larynx is a useful predictor for dysphagia [24], and our study demonstrated that the BDC technique could reduce the mean dose to larynx by approximately 2–3 Gy. Sparing larynx to lower mean dose would reduce the risk of subsequent dysphagia and aspiration, which may affect the treatment compliance of NPC patients during the radiotherapy course and are critical for the quality of life of the patients with long-term survival [1]. Moreover, the BDC technique could reduce the mean dose to oral cavity by 1–2 Gy and is thus potentially beneficial for reducing the incidence of radiotherapy-induced oral mucositis [25,26].

Furthermore, the planning time could be reduced by the BDC technique by 28%, which means that the BDC technique can achieve better planning efficiency. The LDC technique is always time-consuming due to the requirements of repetitive contouring of hot- or cold-spot structures and multiple re-optimization procedures, while only one parameter modification and one or two re-optimization procedures are needed in the BDC technique. The improvement of planning efficiency would help to reduce the heavy routine workloads, as well as reducing the time that the patients must wait for the start of treatment and thus relieving their anxieties.

Conventionally, the base dose function is utilized for optimizing a second plan (top dose plan), such as a boost plan, while considering the first plan (base dose plan), to achieve an optimal plan sum in the optimizer but not in the deliverable pattern with finally calculated dose. However, the base dose function is applied in a new way in the BDC technique, where it is adopted to achieve an optimal second plan (top dose plan) but not a plan sum, in the deliverable pattern but not in the optimizer. In principle, the base dose function is utilized to compensate for the OCE. If an OCE introduces hot/cold spots into the finally calculated dose distribution for substantive tissues/air cavities in the original plan (base dose plan), the second plan (top dose plan) will generate cold/hot spots in the corresponding locations to even out the original hot/cold spots, respectively. After the final dose calculation, by the effect of the OCE again, the cold/hot-spot doses in the optimizer of the second plan (top dose plan) will approach the specified objectives.

The OCE originates from several major sources, as described by Dogan et al [10], including tissue heterogeneity, multi-leaf collimator (MLC) modulation and the optimization algorithm. Possible solutions to the OCE were investigated in a number of researches. The LDC technique, described by Süss et al [12,13], which is helpful for reducing the OCE, is only locally effective in the dose-controlling region. Also, it is a “trial and error” approach because manual adjustments are required for additional constraints. On the contrary, the BDC method is globally effective throughout the entire treatment region and is a systematic approach. The Direct Aperture Optimization (DAO) technique [27–29], which accounts for the series of deliverable MLC apertures in the optimizer, can eliminate the error arising from MLC modulation. Unfortunately, this technique is not available in non-DAO treatment planning systems, such as Eclipse version 10.0, whereas the BDC technique is commonly available because a base dose function or a similar function is a basic feature provided in treatment planning systems. Verbakel et al [30] optimized IMRT plans by dividing PTV into a low-density region with a higher-dose objective setting and a relatively high-density region with a normal-dose objective setting. This approach minimizes only one source of the OCE, that is, the error arising from tissue heterogeneity. It becomes complex when applied to NPC, which has three PTVs. Zacarias and Mill [31] also utilized the base dose function to overcome the OCE. That approach is not the same as ours, because it required a complicated process and software and thus increased the planning steps and time. By contrast, our technique is much simpler and more practical for routine use.

However, there is a limitation in this study. We only investigated the dosimetric outcomes of the recommended technique, and whether it can bring real benefits to patients is still unknown. The actual clinical benefits need to be explored in our further studies.

## Conclusion

The BDC planning technique not only improves the dose conformity and homogeneity of the target, but also spares most OARs. Thus, it may increase the therapeutic ratio of the IMRT for nasopharyngeal cancer. In addition, it offers better planning efficiency. Therefore, the introduced BDC planning technique is recommended for incorporation into the routine clinical practice for the radiotherapy of nasopharyngeal cancer.
